# Cardiometabolic outcomes of once-weekly IcoSema in adults with type 2 diabetes: systematic review and meta-analysis of the COMBINE trials

**DOI:** 10.1186/s40842-026-00333-0

**Published:** 2026-09-02

**Authors:** Adir Alper, Adina Fagin, Aditya Karthikeyan, Mathias Lerner, Adam Gershon, Ahmed Ashraf Morgan, Gal Rubinstein, Raksheeth Agarwal, Robert Faillace

**Affiliations:** Jacobi Medical Center, AECOM, 1400 Pelham Parkway South, Bronx, NY 10461 USA

**Keywords:** IcoSema, Type 2 diabetes, ASCVD risk, HbA1c, Weight reduction, Blood pressure, Lipid profile

## Abstract

**Background:**

Patients with type 2 diabetes face a high residual risk of atherosclerotic cardiovascular disease (ASCVD) despite advances in therapy. Once-weekly IcoSema, a fixed-ratio combination of basal insulin icodec and semaglutide, offers the potential to simultaneously address glycemic control, weight, and multiple cardiometabolic risk factors with a single weekly injection.

**Methods:**

We conducted a PRISMA-compliant systematic review and random-effects meta-analysis of randomized trials from the COMBINE program. Data from COMBINE 1 (active comparator: once-weekly insulin icodec) and COMBINE 3 (active comparator: basal-bolus insulin therapy) (*N* = 1,970) were pooled comparing IcoSema with insulin-based intensification strategies in patients inadequately controlled on basal insulin. COMBINE 2 was excluded because its semaglutide monotherapy comparator addresses a fundamentally different clinical question (escalation from GLP-1 RA monotherapy). The primary focus was on changes in body weight, Systolic blood pressure and HbA1c; key secondary outcomes included changes in lipid profile relevant to ASCVD risk. Certainty of evidence was assessed using GRADE.

**Results:**

IcoSema showed no statistically significant difference in HbA1c reduction compared with control (pooled MD -0.37%, 95% CI -0.95 to 0.21; *P* = 0.21; I²=98%) but shows highly significant body weight reduction (pooled MD -6.10 kg, 95% CI -7.21 to -5.00; *P* < 0.00001). It significantly lowered systolic blood pressure (MD -2.45 mmHg, 95% CI -3.52 to -1.38; *P* < 0.00001), total cholesterol (ETR 0.97, 95% CI 0.95–0.98; *P* = 0.0001), LDL-C (ETR 0.94, 95% CI 0.90–0.98; *P* = 0.005), triglycerides (ETR 0.94, 95% CI 0.91–0.97; *P* = 0.0006), and VLDL-C (ETR 0.94). An exploratory, hypothetical ASCVD risk modeling analysis based on these surrogate-marker changes is presented in the Supplementary Appendix and is intended as an illustration only.

**Conclusions:**

Once-weekly IcoSema delivers meaningful improvements in weight, blood pressure, and atherogenic lipids, all key modifiable drivers of ASCVD in adults with type 2 diabetes. These surrogate benefits, combined with reduced injection burden and low risk of hypoglycemia, suggest potential for enhanced ASCVD prevention and improved long-term adherence. Results should be interpreted as hypothesis-generating only because only two trials met the inclusion criteria. Dedicated cardiovascular outcome trials are essential to validate these surrogate-marker benefits and to confirm whether they translate into a reduction in cardiovascular events.

**Supplementary Information:**

The online version contains supplementary material available at https://doi.org/10.1186/s40842-026-00333-0.

## Introduction

Type 2 diabetes mellitus is a significant health issue, impacting over 500 million adults globally and standing as a major contributor to cardiovascular problems and deaths [1]. Despite therapeutic advances, achieving glycemic targets while minimizing treatment burden, hypoglycemia, and weight gain remains challenging, particularly as the disease progresses and insulin intensification becomes necessary [2]. Traditional basal-bolus therapy provides effective glucose control but requires multiple daily injections and often leads to weight gain and hypoglycemia, which reduces adherence and quality of life [3, 4]. The combination of basal insulin with glucagon-like peptide-1 receptor agonists (GLP-1 RAs) addresses multiple therapeutic goals simultaneously through complementary mechanisms [4, 5]. Fixed-ratio combinations such as insulin degludec/liraglutide and insulin glargine/lixisenatide demonstrate superior glycemic efficacy with reduced hypoglycemia and favorable weight profiles compared to intensified insulin regimens [5, 6]. Meta-analyses show insulin-GLP-1 RA combinations reduce HbA1c by 0.47–0.56% compared to other injectable treatments, with a significantly lower risk of hypoglycemia (RR 0.66) and reduced weight (mean difference − 4.7 kg) when compared with basal-bolus therapy [6]. Real-world evidence confirms these benefits extend to routine clinical practice, with improvements in glycemic control, weight stability, and low hypoglycemia rates across diverse patient populations [7]. However, existing fixed-ratio combinations require daily administration, potentially limiting adherence among patients who prefer fewer injections [8, 9]. Reducing injection frequency represents a critical unmet need in diabetes management. Once-weekly formulations improve medication adherence by approximately 11% compared to daily formulations across multiple therapeutic classes [10]. Insulin icodec, a novel basal insulin with a half-life of approximately one week, has demonstrated non-inferiority to superiority in glycemic control versus once-daily basal insulins in the ONWARDS trials [11, 12]. In ONWARDS 1, once-weekly icodec achieved superior HbA1c reduction (-1.55% vs. -1.35%, *p* = 0.02) and greater time in range (71.9% vs. 66.9%, p0.001) compared to insulin glargine U100 [12]. GLP-1 RAs provide cardiovascular and renal protection beyond glucose lowering through multiple mechanisms, including blood pressure reduction, improved lipid profiles, anti-inflammatory effects, and weight loss [13, 14]. Meta-analyses of cardiovascular outcome trials demonstrate that GLP-1 RAs reduce major adverse cardiovascular events by 13–14%, cardiovascular mortality by 12–13%, and heart failure hospitalization by 11–14% [15, 16]. Injectable semaglutide demonstrated a 26% reduction in major adverse cardiovascular events (HR 0.74, 95% CI 0.58–0.95) in SUSTAIN-6, with oral semaglutide confirming a 14% reduction (HR 0.86, 95% CI 0.77–0.96) in the SOUL trial [14]. These cardiovascular benefits are preserved when GLP-1 RAs are combined with SGLT2 inhibitors, suggesting complementary mechanisms of action [17]. IcoSema represents the first once-weekly fixed-ratio combination of basal insulin and a GLP-1 RA, combining insulin icodec (700 U/mL) with semaglutide (2 mg/mL) in a single injection [18–20]. This formulation reduces annual injections from at least 365 to 52 while leveraging the complementary mechanisms of basal insulin for fasting glucose control and GLP-1 RA for postprandial glucose regulation, weight management, and cardiovascular protection. The COMBINE phase 3a program comprises three 52-week, multinational, randomized, open-label, treat-to-target trials evaluating IcoSema across different clinical scenarios in adults with type 2 diabetes.

COMBINE 1 (*n* = 1,291) compared once-weekly IcoSema to once-weekly insulin icodec in patients inadequately controlled on daily basal insulin, demonstrating superiority for HbA1c reduction (-1.65% vs. -1.39%, estimated treatment difference − 0.26%, p0.0001), body weight change (-3.52 kg vs. + 0.37 kg, p0.0001), time in range during weeks 48–52 (72.9% vs. 65.9%, p0.0001), and lower rates of clinically significant or severe hypoglycemia (0.21 vs. 0.39 episodes per person-year, *p* = 0.0001) [20]. COMBINE 2 (*n* = 683) evaluated IcoSema versus once-weekly semaglutide 1.0 mg in patients inadequately managed on GLP-1 RA therapy, showing superiority for HbA1c reduction (-1.35% vs. -0.90%, estimated treatment difference − 0.44%, p0.0001) and fasting plasma glucose reduction (-2.48 vs. -1.43 mmol/L, *p* = 0.0001), with similar rates of clinically significant or severe hypoglycemia (0.042 vs. 0.036 episodes per person-year, *p* = 0.66) but greater weight gain (+ 0.84 kg vs. -3.70 kg, *p* = 0.0001) [18]. COMBINE 3 (*n* = 679) assessed IcoSema versus basal-bolus therapy (once-daily insulin glargine U100 plus 2–4 daily insulin aspart injections) in patients inadequately controlled on daily basal insulin, demonstrating non-inferior HbA1c reduction (-1.47% vs. -1.40%) with superiority for body weight change (-6.72 kg difference, *p* = 0.0001), lower weekly total insulin dose (-270 U, *p* = 0.0001), and dramatically reduced clinically significant or severe hypoglycemia (0.21 vs. 2.23 episodes per person-year, estimated rate ratio 0.09, *p* = 0.0001) [19].

This systematic review and meta-analysis evaluates the cardiometabolic outcomes of once-weekly IcoSema by pooling data from the COMBINE 1 and 3 trials, focusing on glycemic control, body weight, blood pressure, and lipid profile in adults with type 2 diabetes.

## Methods

### Protocol and reporting guidelines

This systematic review and meta-analysis adhered to the guidelines outlined in the Preferred Reporting Items for Systematic Reviews and Meta-Analyses (PRISMA) 2020 statement [21]. The detailed protocol was prospectively registered in the International Prospective Register of Systematic Reviews (PROSPERO; registration number: CRD420261359075).

### Eligibility criteria (PICO)

The inclusion and exclusion criteria were established a priori based on the PICO (Population, Intervention, Comparator, Outcomes) framework: Population (P): Adult participants (≥ 18 years of age) with a diagnosis of type 2 diabetes mellitus (T2D) or overweight/obesity (Body Mass Index ≥ 27 kg/m² with comorbidity or ≥ 30 kg/m²). Intervention (I): Treatment arms evaluating any dose of Icosema (once-weekly fixed-ratio combination of basal insulin icodec and semaglutide). Comparator (C): Placebo or active control arms (e.g., insulin icodec alone, semaglutide alone, daily basal-bolus insulin, or other standard-of-care therapies). Outcomes (O): Trials must report on at least one of the primary or secondary outcomes defined below. Study Design: Phase 2, and Phase 3 Randomized Controlled Trials (RCTs) published as full-text articles (including data from the COMBINE 1, 2, and 3 program and any earlier phase 1/2 dose-finding or proof-of-concept RCTs). We excluded observational studies, case series, non-randomized trials, and conference abstracts without a corresponding full-text publication. Although our pre-specified protocol allowed both placebo and active comparators, COMBINE 2 was excluded from the quantitative synthesis because its semaglutide monotherapy comparator is non-insulin and addresses a fundamentally different clinical question (escalation from GLP-1 RA monotherapy) than COMBINE 1 and COMBINE 3, which both compare IcoSema with insulin-based regimens (insulin icodec and basal-bolus, respectively). Pooling all three trials would have introduced unacceptable clinical heterogeneity in the comparator arm.

### Outcomes

#### Primary outcomes

1. Efficacy: Percent Change in Body Weight and Change in Systolic Blood Pressure (SBP), Diastolic Blood Pressure, Glycated Hemoglobin (HbA1c) from baseline to the end of the trial period.

#### Key secondary outcomes

1. Cardiovascular Risk Markers: Change in Total Cholesterol, Low-Density Lipoprotein Cholesterol (LDL-C), High-Density Lipoprotein Cholesterol (HDL-C), Very Low-Density Lipoprotein Cholesterol (VLDL-C), Fatty acids, and Triglycerides.

### Search strategy and data sources

A systematic search was conducted across the following electronic databases: MEDLINE/PubMed, Embase (Ovid), Cochrane Central Register of Controlled Trials (CENTRAL), and Web of Science.

The search was supplemented by manual screening of ClinicalTrials.gov (including all COMBINE 1/2/3 registrations) and the reference lists of all included articles and relevant systematic reviews to identify any additional eligible trials. The final search date was March 2026, and no language restriction was applied.

The final search strategy utilized a combination of controlled vocabulary (MeSH and Emtree terms) and text words: (Icosema OR IcoSema OR “insulin icodec semaglutide” OR “icodec AND *semaglutide combination”)* AND *(clinical trial[Publication Type]* OR random*[Title/Abstract] OR *placebo[Title/Abstract]* OR *trial[Title/Abstract]* OR *study[Title/Abstract])*.

### Study selection and data extraction

#### Study selection

All identified records were imported into a reference management software (Zotero, version 7; Corporation for Digital Scholarship), and duplicates were removed. Two independent reviewers (R1 and R2), blind to each other’s decisions, performed the screening process: (1) Title and Abstract Screening, and (2) Full-Text Review against the predefined eligibility criteria. Disagreements were resolved through consensus or by consultation with a third senior reviewer (R3).

#### Data extraction

A standardized data extraction form was developed a priori and applied independently by two reviewers (R1 and R2), with discrepancies resolved by consensus. Extracted variables included study characteristics, patient baseline demographics, intervention details, and outcome data (mean change from baseline, standard deviation, and event counts [22]. Because the analysis relied on aggregate, published trial-level data, there were no missing participant-level data at the level of this meta-analysis; for each outcome we extracted the end-of-trial treatment effects reported by the source trials, which were themselves derived under the trials’ pre-specified estimands (with multiple imputation for missing data performed within the original trials). No additional imputation was undertaken, and outcomes not reported in a form amenable to pooling were not pooled.

### Risk of bias and certainty assessment

#### Risk of bias (RoB)

The methodological quality of all included RCTs was independently assessed by R1 and R2 using the Cochrane Risk of Bias Tool, version 2.0 (RoB 2.0) [23]. Each study received an overall rating of ‘Low risk,’ ‘High risk,’ or unknown risk of bias across five domains (Table S3).

#### Certainty of evidence

The certainty of the evidence for the pooled estimate of each primary and key secondary outcome was assessed using the Grading of Recommendations Assessment, Development and Evaluation (GRADE) approach [24], evaluating risk of bias, inconsistency, indirectness, imprecision, and publication bias.

### Data synthesis and statistical analysis

#### Statistical methods

Meta-analysis performed using a Random-Effects Model (DerSimonian-Laird method) to generate pooled effect estimates, and clinical heterogeneity. Dichotomous Outcomes pooled using the Risk Ratio (RR) with 95% Confidence Intervals (CI). Continuous Outcomes pooled using the Mean Difference (MD) or the Standardized Mean Difference (SMD), presented with 95% CIs. All primary analyses (random-effects model with DerSimonian-Laird estimator, REML for τ², and Wald-type 95% CIs) were pre-specified in the PROSPERO protocol (CRD420261359075). The exploratory ASCVD risk modeling analysis was post hoc and is reported as such in the Discussion.

#### Heterogeneity and robustness

Statistical heterogeneity was quantified using the I^2^ statistic (with I^2^ > 50% indicating substantial heterogeneity) and the chi-square (χ²) test, with a significance threshold of *p* < 0.10. Sensitivity analyses were prespecified to exclude high-risk-of-bias studies and to compare fixed- and random-effects models. With only two trials contributing to each outcome, none of these proved informative, and they are therefore not reported: no trial was rated at high risk of bias, so the exclusion analysis was not applicable; a leave-one-out analysis with k = 2 returns the two trial-specific estimates already displayed in the forest plots; and a fixed-effect model is numerically identical to the random-effects model wherever τ² = 0, while for the two outcomes with substantial heterogeneity (body weight and HbA1c) the fixed-effect assumption of a single common true effect is untenable and would yield a spuriously precise summary. Funnel plots and statistical tests for funnel plot asymmetry (Egger’s and Begg’s tests) were not conducted due to the limited number of included studies (k = 2), as these methods lack sufficient statistical power and visual reliability below the recommended minimum of 10 studies. Given the small number of included trials (k = 2), formal subgroup meta-regression was not feasible. Where substantial heterogeneity was detected (e.g., I²>75%), we therefore report study-level effects narratively and interpret the pooled estimate with caution, acknowledging that the heterogeneity is driven by differences in comparator intensity (insulin icodec in COMBINE 1 vs. basal-bolus therapy in COMBINE 3) rather than by methodological inconsistency. A leave-one-out sensitivity assessment was performed by inspecting each study’s individual estimate against the pooled value [25]. With only two studies contributing to each analysis, heterogeneity is estimated imprecisely; a low or zero I² should therefore be read as the absence of detectable inconsistency between two point estimates rather than as confirmation of true homogeneity, since the analysis is underpowered to detect it.

#### Software

Analyses were performed using Review Manager (RevMan 5.4) (The Cochrane Collaboration, 2020) and the meta-analysis packages in R.

## Results

### Study selection and characteristics

**Fig. 1:**
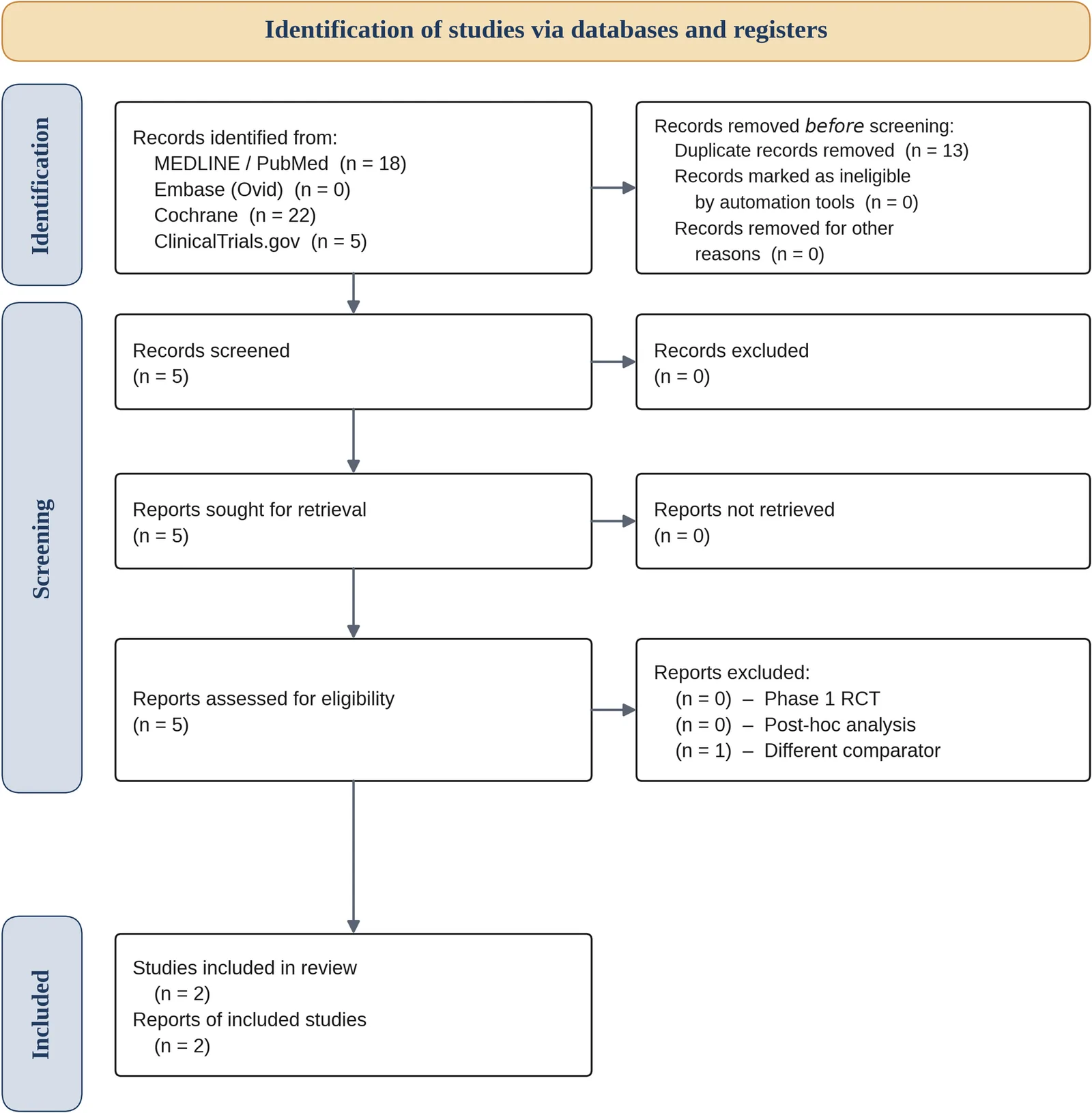
PRISMA 2020 flow diagram of study identification, screening, and inclusion. Of the records identified, two randomized controlled trials (COMBINE 1 and COMBINE 3) met the eligibility criteria and were included in the quantitative synthesis; COMBINE 2 was excluded at full-text review because it used a non-insulin (semaglutide monotherapy) comparator

Two randomized controlled trials met inclusion criteria: Billings et al., 2025 (COMBINE 3) and Mathieu et al., 2025 (COMBINE 1), enrolling 1,970 adults with type 2 diabetes (986 IcoSema; 984 control) (Table S1). COMBINE 2 (Lingvay et al., 2025) was excluded because its semaglutide monotherapy comparator addresses a fundamentally different clinical question (intensification from GLP-1 RA monotherapy in patients not on insulin) than COMBINE 1 and COMBINE 3, which both compare IcoSema with insulin-based regimens. Pooling these three trials would have introduced substantial clinical heterogeneity in the comparator, biasing the pooled estimate of relative treatment effect. We acknowledge that this exclusion limits generalizability to GLP-1 RA-experienced, insulin-naive patients; nonetheless, retaining the comparator-class consistency was essential to the validity of the quantitative synthesis. All outcomes were analyzed using random-effects models with Wald-type confidence intervals and restricted maximum-likelihood estimation for τ². The study identification and selection process is summarized in the PRISMA 2020 flow diagram **(**Fig. 1**).**

### Primary outcomes

#### Blood pressure

**Fig. 2:**
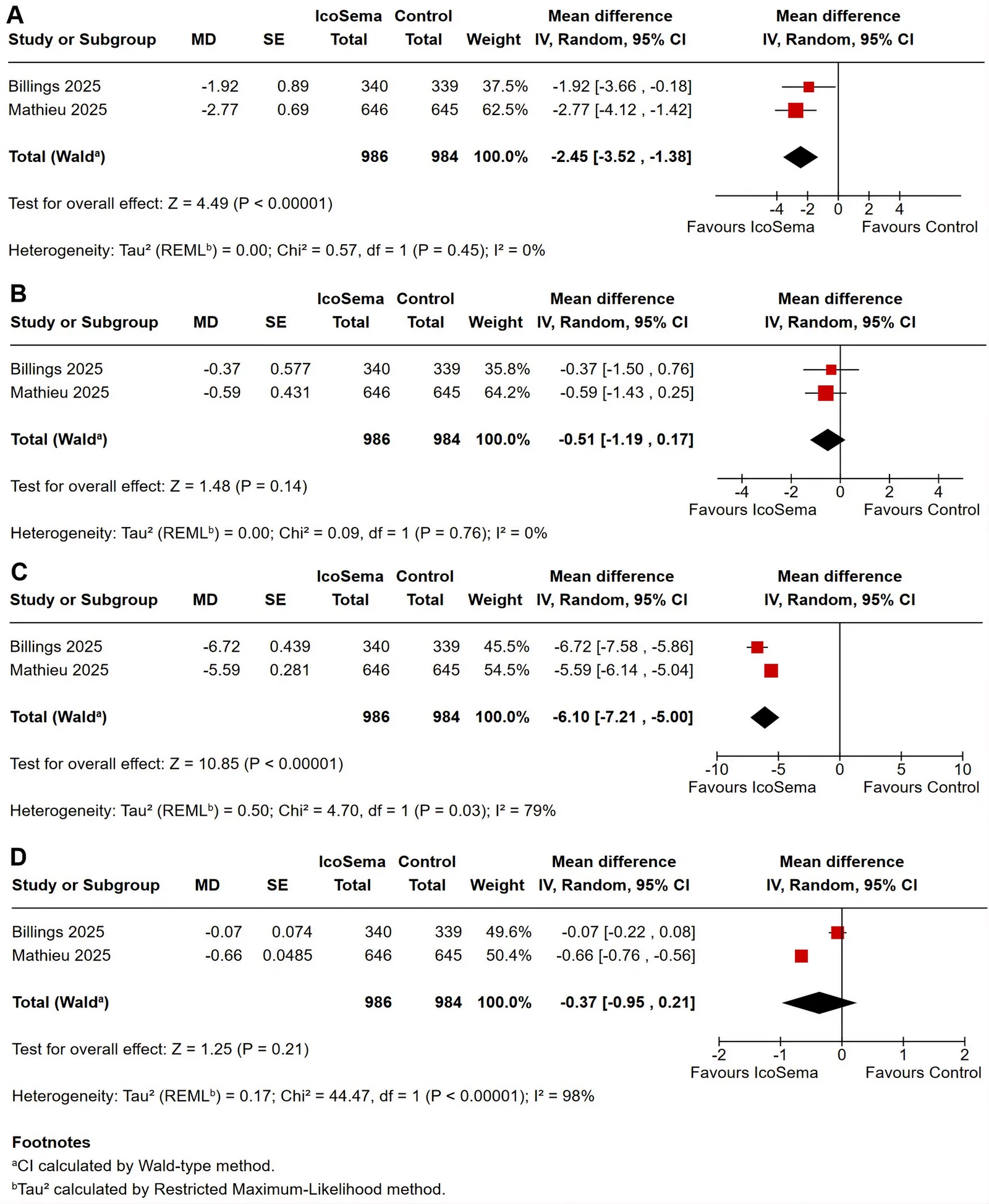
Forest plots of pooled mean differences for systolic and diastolic blood pressure, body weight, and HbA1c with once-weekly IcoSema versus control. **(A)** Systolic blood pressure (mmHg); **(B)** Diastolic blood pressure (mmHg); **(C)** Body weight (kg); **(D)** HbA1c (%). Negative values favor IcoSema. Pooled estimates (diamonds) were derived from inverse-variance random-effects models. τ² estimated by REML; CIs by Wald-type method

Systolic blood pressure was significantly lower with IcoSema (pooled MD -2.45 mmHg, 95% CI -3.52 to -1.38; Z = 4.49, *P* < 0.00001; I² = 0%; Fig. 2A), with consistent effects across both trials. Diastolic blood pressure showed a non-significant trend favoring IcoSema (pooled MD -0.51 mmHg, 95% CI -1.19 to 0.17; Z = 1.48, *P* = 0.14; I² = 0%; Fig. 2B).

#### Body weight

IcoSema significantly reduced body weight compared with control (pooled MD -6.10 kg, 95% CI -7.21 to -5.00; Z = 10.85, *P* < 0.00001; Fig. 2C). Heterogeneity was moderate-to-high (τ² = 0.50, χ² = 4.70, df = 1, *P* = 0.03, I² = 79%), with a larger absolute weight difference in Billings 2025 (MD -6.72 kg) than in Mathieu 2025 (MD -5.59 kg), consistent with the weight-gain effect of the basal-bolus comparator.

#### Glycated hemoglobin (HbA1c)

There was no statistically significant difference in HbA1c reduction between IcoSema and control (pooled MD -0.37%, 95% CI -0.95 to 0.21; Z = 1.25, *P* = 0.21; Fig. 2D). However, the difference was not statistically significant, and heterogeneity was very high (τ² = 0.17, χ² = 44.47, df = 1, *P* < 0.00001, I² = 98%). The effect was driven almost entirely by the larger benefit observed in Mathieu 2025 (MD -0.66%) versus the near-null result in Billings 2025 (MD -0.07%), reflecting the differing comparator intensities. Given this extreme heterogeneity, the pooled HbA1c estimate should not be interpreted as a single summary effect; it is presented for transparency, and the trial-specific results are more informative. Clinically, these data indicate that IcoSema is superior to icodec monotherapy for glycaemic control but comparable to intensive basal-bolus therapy, a clinically meaningful distinction that the pooled “no difference” estimate obscures. Future analyses with additional trials, including subgroup analyses by baseline HbA1c and prior insulin exposure, will be needed to explain this heterogeneity. Accordingly, our interpretation of glycemic efficacy rests on the two trial-specific estimates read as a narrative synthesis, not on the pooled value, which we retain only for completeness.

### Key secondary cardiometabolic outcomes

**Fig. 3:**
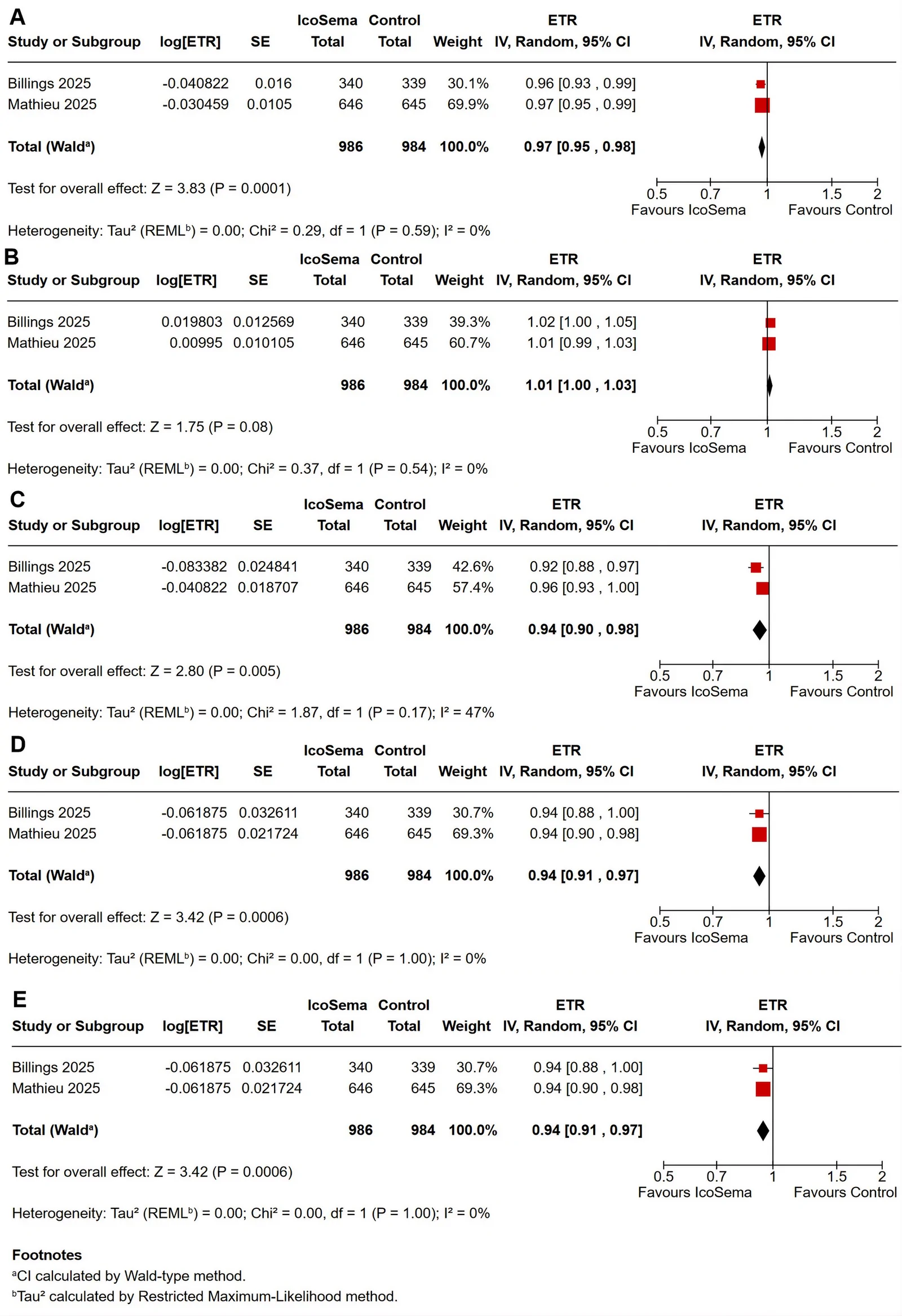
Forest plots of lipid outcomes with IcoSema versus control. **(A)** Total cholesterol; **(B)** HDL cholesterol; **(C)** LDL cholesterol; **(D)** VLDL cholesterol; **(E)** Triglycerides. ETR < 1.0 favors IcoSema. Pooled estimates (diamonds) were derived from inverse-variance random-effects models. τ² estimated by REML; CIs by Wald-type method

*Lipid profile (reported as exponentiated treatment ratios [ETR]*,* equivalent to the ratio of geometric means)*: Total cholesterol was significantly reduced with IcoSema (ETR 0.97, 95% CI 0.95–0.98; Z = 3.83, *P* = 0.0001; I² = 0%; Fig. 3A), corresponding to an approximately 3% relative reduction. HDL cholesterol showed a small, borderline increase (ETR 1.01, 95% CI 1.00-1.03; Z = 1.75, *P* = 0.08; I² = 0%; Fig. 3B). LDL cholesterol was significantly reduced (ETR 0.94, 95% CI 0.90–0.98; Z = 2.80, *P* = 0.005; I² = 47%; Fig. 3C). VLDL cholesterol (ETR 0.94, 95% CI 0.91–0.97; Z = 3.42, *P* = 0.0006; I² = 0%; Fig. 3D) and triglycerides (ETR 0.94, 95% CI 0.91–0.97; Z = 3.42, *P* = 0.0006; I² = 0%; Fig. 3E) were both significantly reduced, with identical point estimates and no between-study heterogeneity. These values translate directly into relative changes: an ETR of 0.94 corresponds to an approximately 6% relative reduction (LDL-C, VLDL-C, and triglycerides), an ETR of 0.97 to an approximately 3% relative reduction (total cholesterol), and an ETR of 1.01 to an approximately 1% relative increase (HDL-C). The certainty of evidence for each outcome was assessed using GRADE and ranged from low (HbA1c, downgraded for very serious inconsistency) to moderate (all other outcomes); the full GRADE evidence profile, with the explicit certainty rating and rationale for every outcome, is provided in Supplementary Table S2.

## Discussion

The pooled results portray once-weekly IcoSema as a potent cardiometabolic modulator that delivers meaningful, multifaceted benefits beyond what either basal insulin or basal-bolus regimens alone can achieve. The striking 6.1 kg greater weight loss and consistent improvements in blood pressure and atherogenic lipids occurred across two large, treat-to-target trials that deliberately tested IcoSema in the very populations where therapeutic inertia is highest, patients already on insulin or struggling with daily regimens. Remarkably, these improvements were accomplished with just 52 injections a year, compared to 365 or more, which could change the long-term adherence in everyday practice. This expectation is grounded in adherence data across therapeutic classes: a meta-analysis of injectable GLP-1 receptor agonists found significantly higher adherence and persistence with once-weekly than with once-daily dosing, and switching from a daily to a once-weekly regimen has been shown to improve medication adherence and reduce the quantity of unused drug [8, 10]. By consolidating basal insulin and a GLP-1 RA into a single weekly injection, IcoSema may therefore translate this dosing advantage into improved real-world persistence, although adherence was not a measured endpoint in the COMBINE trials and this remains a plausible inference rather than a demonstrated outcome.

Yet the data also invite deeper reflection. The non-significant overall HbA1c difference (-0.37%) masks profound heterogeneity: IcoSema was markedly superior to once-weekly icodec monotherapy (Mathieu 2025) but nearly identical to basal-bolus therapy (Billings 2025). This pattern is biologically coherent - when the comparator already provides aggressive fasting glucose control, the incremental glycemic gain from the semaglutide component is modest, yet the complementary weight-loss and cardiometabolic benefits show persistent efficacy. The same low-heterogeneity signals for lipids, SBP, and triglycerides suggest these benefits are more “portable” across clinical contexts and less dependent on the exact insulin backbone. The same comparator-dependence applies to body weight: the weight-loss benefit of IcoSema appears partly contingent on comparator choice, being larger against weight-gaining basal-bolus therapy (Billings 2025, MD -6.72 kg) than against icodec monotherapy (Mathieu 2025, MD -5.59 kg). The 1.13 kg difference between trials is not trivial, and whether the 6.1 kg average benefit would persist against other GLP-1 RA/insulin combinations remains unknown.

To explore how these surrogate-marker changes might map onto cardiovascular risk, we performed an exploratory, hypothetical risk-modeling exercise that applied the pooled treatment effects to the 2013 ACC/AHA Pooled Cohort Equations; the full analysis, including its assumptions and limitations, is now provided in the Supplementary Appendix (Supplementary Methods and Table S4). This modeling is hypothetical and illustrative only, is not intended as a clinical prediction, does not replace dedicated cardiovascular outcome trials, and should not be used to inform clinical decision-making [26, 27].

IcoSema therefore occupies a unique therapeutic niche: it is not merely “another GLP-1 RA-insulin combination” but the first agent that compresses the entire cardiometabolic toolkit-glycemic control, weight management, lipid improvement, and blood-pressure lowering-into one weekly dose. The present analysis extends the findings of Kamrul-Hasan et al. (2025) [28] who conducted a prior meta-analysis of IcoSema RCTs focusing on HbA1c and body weight; our work adds the first pooled estimates of blood pressure and atherogenic lipid outcomes alongside the ASCVD risk modeling. In an era when injection fatigue remains a silent driver of therapeutic failure, this pharmacokinetic elegance may prove as transformative as the molecules themselves [29, 30]. Larger cardiovascular outcome trials are still needed to confirm hard-event reduction, yet the surrogate data presented here already paint an intriguing picture: a once-weekly injection that quietly resets multiple drivers of atherosclerosis while simultaneously easing the daily burden of diabetes self-management. For millions of patients and their clinicians, that combination may represent the most compelling advance in insulin-based therapy in decades.

The blood-pressure and lipid benefits observed here are mechanistically plausible and consistent with the known pharmacology of the GLP-1 receptor agonist component. GLP-1 receptor agonists lower systolic blood pressure through several complementary pathways, including natriuresis, improvements in endothelial function and arterial compliance, reductions in vascular inflammation, and the haemodynamic consequences of weight loss [13, 14]. Their effect on atherogenic lipids is similarly multifactorial: reduced hepatic VLDL production and secretion, delayed gastric emptying that blunts postprandial lipemia, and weight-loss-mediated improvements in insulin sensitivity together act to lower total cholesterol, LDL-C, VLDL-C, and triglycerides [14]. The basal-insulin component contributes little independent benefit to blood pressure or lipids, so the cardiometabolic signal observed here is most plausibly attributable to the semaglutide component and to the weight loss it drives.

Although the present synthesis focused on cardiometabolic efficacy, the safety profile is integral to the clinical value of IcoSema. In the pooled trials, IcoSema was associated with low rates of clinically significant or severe hypoglycaemia: 0.21 versus 0.39 episodes per person-year against once-weekly icodec in COMBINE 1, and 0.21 versus 2.23 episodes per person-year against basal-bolus therapy in COMBINE 3 (estimated rate ratio 0.09) [19, 20]. This favourable hypoglycaemia profile, achieved alongside superior or non-inferior glycaemic control, is one of the most clinically meaningful features of the regimen. As expected for a GLP-1 receptor agonist-containing therapy, gastrointestinal adverse events (most commonly nausea, diarrhoea, vomiting, and decreased appetite) were the most frequent treatment-emergent events; these were predominantly mild to moderate, occurred chiefly during dose escalation, and were generally transient. We did not pool hypoglycaemia or gastrointestinal events quantitatively because only two trials, with differing comparators and event-reporting conventions, were available; nonetheless, these data indicate that the cardiometabolic benefits described above are not offset by an unfavourable tolerability or hypoglycaemia profile.

Several limitations warrant consideration. The most important limitation is the very small number of included trials (k = 2). With only two studies, the pooled estimates are inherently fragile, between-study variance (τ²) is poorly estimated, formal subgroup or meta-regression analyses are not feasible, and standard tools for assessing publication bias (funnel plots, Egger’s test) cannot be applied. The pooled effects should therefore be regarded as preliminary and hypothesis-generating rather than definitive. A concurrent meta-analysis by Kamrul-Hasan et al. (2025) reported overlapping HbA1c and weight findings; our work extends that evidence to blood pressure and atherogenic lipid outcomes but inherits the same fundamental constraint of an underpowered evidence base [28]. First, only two of the three COMBINE trials contributed data to the pooled analyses; exclusion of COMBINE 2 limits the generalizability of findings to patients escalating from GLP-1 RA monotherapy. Additionally, while both trials used an insulin-based comparator class, the specific comparators were different, COMBINE 1 used once-weekly insulin icodec, whereas COMBINE 3 utilized basal-bolus therapy; this residual clinical heterogeneity is the most likely driver of the very high I² observed for HbA1c (98%), and pooled estimates for that outcome should be interpreted with particular caution. These findings do not apply to GLP-1 RA-naive, insulin-naive patients, nor to the growing population escalating from GLP-1 RA monotherapy that was represented by the excluded COMBINE 2 trial. In COMBINE 2 IcoSema produced a greater HbA1c reduction than semaglutide monotherapy (-1.35% vs. -0.90%) but was accompanied by modest weight gain rather than the weight loss seen with semaglutide (+ 0.84 kg vs. -3.70 kg), a pattern that differs from the insulin-comparator setting analyzed here and underscores why pooling across comparator classes was avoided. A future three-way meta-analysis incorporating all COMBINE trials using comparator-adjusted methods may help to address this gap. Second, both source trials used an open-label design, which introduces the possibility of performance and detection biases, particularly for subjective outcomes. Because participants and investigators were aware of allocation, knowledge of receiving the novel agent could plausibly influence diet, physical activity, and titration behavior; for the objective, centrally measured endpoints emphasized here (weight, blood pressure, and laboratory lipids) any such bias is likely to be small, but for hypoglycemia and adverse-event reporting detection bias could lead to underestimation of true event rates. The direction and magnitude of this bias cannot be quantified from the available data, and only blinded comparators, or at minimum blinded outcome assessment, in future trials would definitively resolve it. Third, all cardiovascular outcomes reported here are surrogate markers (blood pressure, lipids); no dedicated cardiovascular outcome trial for IcoSema has been completed, and hard-event reduction remains to be confirmed. To our knowledge, no cardiovascular outcome trial powered for major adverse cardiovascular events has yet been registered for the IcoSema fixed-ratio combination, and none is expected to report in the near term; for now, any cardiovascular expectation rests on indirect evidence from the semaglutide component, whose benefit is established in SUSTAIN-6 and SOUL, rather than on the combination product itself. Until such a trial is conducted, the present surrogate findings should be treated as generating hypotheses for that future work. Fourth, the ASCVD risk modeling using the 2013 ACC/AHA Pooled Cohort Equations is illustrative and exploratory; the equations are not formally validated for treatment-effect modeling, were derived primarily in non-diabetic populations and may over- or under-estimate absolute risk in type 2 diabetes. Fifth, both trials were industry-sponsored by Novo Nordisk, which may introduce sponsorship bias, although both were published in high-impact peer-reviewed journals and used independent statistical analysis. Beyond providing funding, the sponsor was involved in the design, conduct, and analysis of both source trials, and such involvement in trial design, data analysis, and the decision to publish is a recognized potential source of sponsorship bias; by contrast, the present meta-analysis was investigator-initiated, received no external funding, and was conducted independently of the sponsor. The 52-week follow-up may be insufficient to capture long-term cardiometabolic trends or late safety signals.

## Conclusion

IcoSema, when taken just once a week, led to a notable weight loss of 6.1 kg and showed improvements in important cardiometabolic markers like systolic blood pressure, total cholesterol, LDL-C, and triglycerides, especially when compared to control treatments in adults with type 2 diabetes. These positive shifts in cardiovascular risk factors, achieved with just one injection each week, make IcoSema an attractive option for streamlining treatment and possibly lowering the long-term risk of atherosclerotic cardiovascular disease (ASCVD). Because the evidence base comprises only two trials, these findings should be regarded as preliminary and hypothesis-generating and interpreted cautiously pending a larger evidence base. Dedicated cardiovascular outcome trials are essential to validate these surrogate benefits and to confirm whether they translate into a reduction in clinical events.

## Supplementary Information

Below is the link to the electronic supplementary material.


Supplementary Material 1


## Data Availability

No datasets were generated or analysed during the current study.
