# Supplementary material for: Cardiometabolic outcomes of once-weekly IcoSema in adults with type 2 diabetes: systematic review and meta-analysis of the COMBINE trials
Source: Cardiovasc Diabetol Endocrinol Rep. 2026 Sep 2;12:59. doi: 10.1186/s40842-026-00333-0 (PMC13536828; doi:10.1186/s40842-026-00333-0)
Supplement: Supplementary file 1 [file 40842_2026_333_MOESM1_ESM.docx]

*Systematic Review and Meta-Analysis*

Cardiometabolic Outcomes of Once-Weekly IcoSema in Adults with Type 2 Diabetes: Systematic Review and Meta-Analysis of the COMBINE Trials

**Supplementary Appendix**

# Supplementary Tables

## Table S1. Characteristics of Included Randomized Controlled Trials

| **Trial (Registration)** | **Design** | **Duration** | **Population** | **N (I/C)** | **Intervention** | **Comparator** | **Mean Age, y** | **Male, %** | **Baseline HbA1c, % (SD)** |
| --- | --- | --- | --- | --- | --- | --- | --- | --- | --- |
| COMBINE 1 Mathieu et al., 2025 Lancet Diabetes Endocrinol (NCT05352815) | Open-label, treat-to-target, randomized, phase 3a, superiority | 52 weeks (+ 5-wk follow-up) | Adults with T2D inadequately controlled on daily basal insulin (20–80 U/day) ± OGLMs; HbA1c 7.0–10.0% | 1291 (646 / 645) | Once-weekly IcoSema (icodec 700 U/mL + semaglutide 2 mg/mL); starting dose 40 steps; max 350 steps | Once-weekly insulin icodec 700 U/mL (no max dose) | 60.6 (10.3) | 62% | 8.22 (0.84) |
| COMBINE 2 Lingvay et al., 2025 Diabetologia (NCT05259033) | Open-label, parallel-group, randomized, phase 3a, superiority | 52 weeks (+ 5-wk follow-up) | Adults with T2D inadequately managed on GLP-1 RA therapy ± OGLMs; HbA1c 7.0–10.0%; insulin-naive | 683 (342 / 341) | Once-weekly IcoSema; starting dose 40 steps; max 350 steps | Once-weekly semaglutide 1.0 mg (fixed-dose escalation: 0.25 → 0.5 → 1.0 mg) | 59.1 (10.2) | 58% | 8.00 (0.75) |
| COMBINE 3 Billings et al., 2025 Lancet Diabetes Endocrinol (NCT05013229) | Open-label, treat-to-target, non-inferiority, randomized, phase 3a | 52 weeks (+ 5-wk follow-up) | Adults with T2D inadequately controlled on daily basal insulin (20–80 U/day) ± OGLMs; HbA1c 7.0–10.0%; GLP-1 RA-naive | 679 (340 / 339) | Once-weekly IcoSema; starting dose 40 steps; max 350 steps | Basal-bolus therapy: once-daily insulin glargine U100 + 2–4 daily insulin aspart injections | 59.6 (10.4) | 59% | 8.30 (0.82) |

**Table 1 (continued). Additional Baseline Characteristics**

| **Trial** | **BMI, kg/m² (SD)** | **Diabetes Duration, y (SD)** | **Bodyweight, kg (SD)** | **FPG, mmol/L (SD)** | **Metformin, %** | **SGLT2i, %** | **White, %** | **Countries / Sites** |
| --- | --- | --- | --- | --- | --- | --- | --- | --- |
| **COMBINE 1** | 29.9 (4.7) | 15.3 (7.8) | 84.5 (17.1) | 8.6 (2.9) | 84% | 39% | 63% | 20 / 192 |
| **COMBINE 2** | 31.1 (4.7) | 12.6 (6.9) | 89.2 (18.0) | 9.5 (2.7) | 88% | 44% | 64% | 13 / 121 |
| **COMBINE 3** | 30.4 (5.0) | 14.4 (7.6) | 85.8 (17.8) | 8.7 (2.8) | 86% | 38% | 53% | 14 / 109 |

*T2D = type 2 diabetes mellitus; OGLMs = oral glucose-lowering medications; GLP-1 RA = glucagon-like peptide-1 receptor agonist; FPG = fasting plasma glucose; SGLT2i = sodium–glucose cotransporter 2 inhibitor; I/C = intervention/comparator. Values are mean (SD) unless otherwise noted. All trials were funded by Novo Nordisk.*

## Table S2. Summary of Findings and Certainty of Evidence (GRADE)

| **Summary of Findings and Certainty of Evidence (GRADE)** | | | | | | | | | | |
| --- | --- | --- | --- | --- | --- | --- | --- | --- | --- | --- |
| *Population: Adults with type 2 diabetes inadequately controlled on daily basal insulin. Intervention: Once-weekly IcoSema. Comparator: Insulin icodec or basal-bolus therapy. Setting: Multinational outpatient clinics (COMBINE 1 and COMBINE 3 pooled; N = 1,970).* | | | | | | | | | | |
| **Outcome (timeframe)** | **No. of Studies** | **No. of Participants** | **Pooled Effect Estimate (95% CI)** | **Risk of Bias** | **Inconsistency** | **Indirectness** | **Imprecision** | **Publication Bias** | **Overall Certainty** | **Interpretation** |
| **Change in HbA1c, % (52 wk)** | 2 | 1,970 | MD -0.37% (95% CI -0.95 to 0.21) P = 0.21; I² = 98% | Not serious ¹ | Very serious ² | Not serious | Serious ³ | Undetected ⁴ | **⊕⊕⊖⊖ LOW** | IcoSema may reduce HbA1c compared with control, but effect is uncertain due to high heterogeneity |
| **Change in body weight, kg (52 wk)** | 2 | 1,970 | MD -6.10 kg (95% CI -7.21 to -5.00) P < 0.00001; I² = 79% | Not serious ¹ | Serious ⁵ | Not serious | Not serious | Undetected ⁴ | **⊕⊕⊕⊖ MODERATE** | IcoSema probably results in a large reduction in body weight compared with control |
| **Change in SBP, mmHg (52 wk)** | 2 | 1,970 | MD -2.45 mmHg (95% CI -3.52 to -1.38) P < 0.00001; I² = 0% | Not serious ¹ | Not serious | Not serious | Not serious | Undetected ⁴ | **⊕⊕⊕⊖ MODERATE** | IcoSema probably results in a small reduction in systolic blood pressure |
| **Change in DBP, mmHg (52 wk)** | 2 | 1,970 | MD -0.51 mmHg (95% CI -1.19 to 0.17) P = 0.14; I² = 0% | Not serious ¹ | Not serious | Not serious | Serious ³ | Undetected ⁴ | **⊕⊕⊕⊖ MODERATE** | IcoSema may result in little to no difference in diastolic blood pressure |
| **Total cholesterol (ETR) (52 wk)** | 2 | 1,970 | ETR 0.97 (95% CI 0.95 to 0.98) P = 0.0001; I² = 0% | Not serious ¹ | Not serious | Not serious | Not serious | Undetected ⁴ | **⊕⊕⊕⊖ MODERATE** | IcoSema probably results in a small reduction in total cholesterol (≈3% relative reduction) |
| **LDL cholesterol (ETR) (52 wk)** | 2 | 1,970 | ETR 0.94 (95% CI 0.90 to 0.98) P = 0.005; I² = 47% | Not serious ¹ | Not serious ⁶ | Not serious | Not serious | Undetected ⁴ | **⊕⊕⊕⊖ MODERATE** | IcoSema probably results in a moderate reduction in LDL-C (≈6% relative reduction) |
| **Triglycerides (ETR) (52 wk)** | 2 | 1,970 | ETR 0.94 (95% CI 0.91 to 0.97) P = 0.0006; I² = 0% | Not serious ¹ | Not serious | Not serious | Not serious | Undetected ⁴ | **⊕⊕⊕⊖ MODERATE** | IcoSema probably results in a reduction in triglycerides (≈6% relative reduction) |
| **HDL cholesterol (ETR) (52 wk)** | 2 | 1,970 | ETR 1.01 (95% CI 1.00 to 1.03) P = 0.08; I² = 0% | Not serious ¹ | Not serious | Not serious | Serious ³ | Undetected ⁴ | **⊕⊕⊕⊖ MODERATE** | IcoSema may result in little to no change in HDL-C |
| **GRADE Certainty Ratings: ⊕⊕⊕⊕ HIGH; ⊕⊕⊕⊖ MODERATE; ⊕⊕⊖⊖ LOW; ⊕⊖⊖⊖ VERY LOW**  *CI = confidence interval; DBP = diastolic blood pressure; ETR = estimated treatment ratio (ratio of geometric means); MD = mean difference; SBP = systolic blood pressure.*  **Footnotes:**  ¹ Both trials were open-label, which introduces risk of performance and detection bias. However, primary outcomes (HbA1c, weight, BP, lipids) are objective laboratory-measured endpoints minimally affected by knowledge of allocation. Not downgraded.  ² I² = 98%; marked discordance between COMBINE 1 (MD -0.66%) and COMBINE 3 (MD -0.06%) attributable to differing comparator insulin intensity. Downgraded two levels.  ³ 95% CI includes the null (no effect); downgraded one level for imprecision.  ⁴ Too few studies (n = 2) to formally assess publication bias via funnel plots or Egger’s test. Not downgraded but noted as a limitation.  ⁵ I² = 79%; moderate heterogeneity. Direction of effect consistent across both trials. Downgraded one level.  ⁶ I² = 47%; moderate heterogeneity below the pre-specified 50% threshold. Not downgraded.  *All outcomes start at HIGH certainty (RCT evidence) and are downgraded by one level for open-label design not affecting objective outcomes (not applied) or for domain-specific concerns as noted above.* | | | | | | | | | | |

## Table S3. Risk of Bias Assessment (Cochrane RoB 2.0)

| **Study** | **D1: Randomization Process** | **D2: Deviations from Intended Interventions** | **D3: Missing Outcome Data** | **D4: Measurement of the Outcome** | **D5: Selection of the Reported Result** | **Overall** |
| --- | --- | --- | --- | --- | --- | --- |
| **COMBINE 1 (Mathieu et al., 2025)** | **Low risk** | Some concerns | **Low risk** | **Low risk** | **Low risk** | Some concerns |
| **COMBINE 2 (Lingvay et al., 2025)** | **Low risk** | Some concerns | **Low risk** | **Low risk** | **Low risk** | Some concerns |
| **COMBINE 3 (Billings et al., 2025)** | **Low risk** | Some concerns | **Low risk** | **Low risk** | **Low risk** | Some concerns |
| **Risk of Bias Justifications (Cochrane RoB 2.0):**  D1 – Randomization process: All three trials used centralized computer-generated randomization (1:1) via interactive web-response or randomization and trial supply management systems. Baseline characteristics were well-balanced across treatment arms in all trials. Rated: Low risk.  D2 – Deviations from intended interventions: All trials were open-label; participants and investigators were aware of treatment assignment. Although open-label designs may influence behavior (e.g., dietary modifications, dose titration intensity), the treat-to-target protocols standardized titration algorithms, and objective outcome measures (HbA1c, weight, laboratory values) are minimally susceptible to detection bias. Some participants in both arms initiated non-randomized rescue medications. Both intercurrent events were handled by treatment policy strategy (intention-to-treat). Rated: Some concerns due to open-label design.  D3 – Missing outcome data: Completion rates were high in all trials (COMBINE 1: 90–93%; COMBINE 2: 94–96%; COMBINE 3: 86–92%). Missing data were handled via multiple imputation. Sensitivity analyses (tipping point) confirmed robustness. Rated: Low risk.  D4 – Measurement of the outcome: Primary and key secondary outcomes (HbA1c, body weight, FPG, blood pressure, lipids) were measured using validated, standardized laboratory assays and calibrated instruments by central laboratories blinded to treatment allocation. CGM data were blinded. Rated: Low risk.  D5 – Selection of the reported result: All trials were pre-registered on ClinicalTrials.gov with pre-specified statistical analysis plans. Hierarchical testing procedures and primary estimands were defined a priori and reported as pre-specified. No evidence of selective outcome reporting. Rated: Low risk.  **Overall: All three trials received an overall judgment of “Some concerns” due to the open-label design (Domain 2). No trial was rated as high risk of bias.** | | | | | | |

**Supplementary Methods: Exploratory ASCVD Risk Modeling**

We performed a single exploratory, post hoc modeling exercise (not pre-registered in PROSPERO) to illustrate how the pooled surrogate-marker changes might map onto absolute cardiovascular risk. It is hypothetical and illustrative only, is not a clinical prediction, and should not inform clinical decisions.

For a representative index patient (a 60-year-old non-Hispanic white man with type 2 diabetes, non-smoker, no antihypertensive therapy, total cholesterol 190 mg/dL, HDL-C 45 mg/dL, systolic blood pressure 130 mmHg), the 2013 ACC/AHA Pooled Cohort Equations estimated a baseline 10-year ASCVD risk of 19.47%. Applying the pooled treatment effects (systolic blood pressure -2.45 mmHg; total cholesterol × ETR 0.97; HDL-C × ETR 1.01) lowered the estimate to 18.27% an absolute18.27%, an absolute risk reduction of ≈1.19 percentage points over 10 years (Table S4).

This rests on unverified assumptions: the equations were derived in largely non-diabetic populations for primary prediction, not treatment-effect modeling; the exercise assumes linear, additive surrogate changes in a single hypothetical patient; and no hard-endpoint data exist for IcoSema. It should therefore be read as indicating the likely direction and rough scale of effect, not clinical benefit.

**Table S4. Exploratory, hypothetical ASCVD risk modeling for a single representative patient (illustrative only).**

| **Parameter** | **Control (baseline)** | **With IcoSema (modeled)** |
| --- | --- | --- |
| Index patient | 60-year-old non-Hispanic white man with type 2 diabetes; non-smoker; not on antihypertensive therapy | (same individual, treated) |
| Total cholesterol, mg/dL | 190 | ≈184 (applying ETR 0.97) |
| HDL-C, mg/dL | 45 | ≈45 (applying ETR 1.01) |
| Systolic blood pressure, mmHg | 130 | 127.6 (-2.45 mmHg) |
| 10-year ASCVD risk (2013 ACC/AHA Pooled Cohort Equations) | 19.47% | 18.27% |
| Absolute risk reduction over 10 years | — | 1.19 percentage points |

ASCVD = atherosclerotic cardiovascular disease; ETR = exponentiated treatment ratio; HDL-C = high-density lipoprotein cholesterol. This modeling is hypothetical and exploratory, was performed post hoc, and is not intended to inform clinical decision-making.
